# Efficacy of Sub-Gingivally Delivered Propolis Nanoparticle in Non-Surgical Management of Periodontal Pocket: A Randomized Clinical Trial

**DOI:** 10.3390/biom13111576

**Published:** 2023-10-26

**Authors:** Sushree Ambika Sahu, Saurav Panda, Abhaya Chandra Das, Lora Mishra, Satchidananda Rath, Krzysztof Sokolowski, Manoj Kumar, Rinkee Mohanty, Rashmita Nayak, Anurag Satpathy, Barbara Lapinska

**Affiliations:** 1 Department of Periodontics and Oral Implantology, Institute of Dental Sciences, Siksha ‘O’ Anusandhan University, Bhubaneswar 751003, Odisha, India; sahusushreeambika@gmail.com (S.A.S.); abhayadas@soa.ac.in (A.C.D.); manojkumar@soa.ac.in (M.K.); rinkeemohanty@soa.ac.in (R.M.); rashmitanayak@soa.ac.in (R.N.); anuragsatpathy@soa.ac.in (A.S.); 2 Department of Conservative Dentistry & Endodontics, Institute of Dental Sciences, Siksha ‘O’ Anusandhan University, Bhubaneswar 751003, Odisha, India; loramishra@soa.ac.in; 3 Department of Physics, School of Basic Sciences, Indian Institute of Technology, Bhubaneswar 752050, Odisha, India; srath@iitbbs.ac.in; 4 Department of Conservative Dentistry, Medical University of Lodz, 251 Pomorska St., 92-213 Lodz, Poland; krzysztof.sokolowski@umed.lodz.pl; 5 Department of General Dentistry, Medical University of Lodz, 251 Pomorska St., 92-213 Lodz, Poland

**Keywords:** chronic periodontitis, periodontal pocket, local drug delivery, propolis nanoparticle

## Abstract

Naturally sourced products like propolis are commonly employed for the non-surgical treatment of periodontal pockets. The use of nanoparticle formulations of these natural remedies has the potential to improve treatment outcomes. The aim of the present study was to evaluate the efficacy of sub-gingivally delivered propolis nanoparticles in the non-surgical management of periodontal pockets. Forty patients diagnosed with periodontitis presenting at least one periodontal pocket with a probing pocket depth between 4 and 6 mm were selected. Patients were randomly assigned into the control group (*n* = 20), which received scaling and root planing (SRP) and saline (SRP + Saline), and the test group (*n* = 20), which received SRP and sub-gingivally delivered propolis nanoparticles (PRO) into the periodontal pocket (SRP + PRO). The clinical parameters recorded were plaque index (PI), gingival index (GI), relative attachment loss (RAL), probing pocket depth (PPD), and bleeding on probing (BOP). They were assessed at baseline, one month, and three months post therapy. The results indicated that there was a significant improvement in clinical parameters (*p* < 0.05) in the test sites compared with the control sites at the end of the study. The gingival index at one month and three months was found to be significantly better in the SRP + PRO group than the SRP + Saline group, with a *p* value of <0.001. The BOP, PPD, and RAL showed significant improvement with the SRP + PRO group at the end of the 3-month follow-up with *p* values of 0.0001, 0.001, and 0.05, respectively. The subgingival delivery of propolis nanoparticles showed promising results as an adjunct to SRP in patients with periodontitis presenting periodontal pockets.

## 1. Introduction

Periodontal disease [PD] is an immuno-inflammatory destructive disease of periodontal tissues characterized by the loss of soft tissue attachment and alveolar bone loss caused by pathogenic microorganisms resulting in pocket formation and/or gingival recession [1]. Periodontal disease is one of the most common chronic infectious diseases among adults and is caused by the accumulation of bacterial biofilm [1,2].

The periodontal pocket is a pathologically deepened gingival sulcus caused by the apical migration of junctional epithelium. The coronal movement of the gingival margin without the destruction of underlying periodontal tissues creates a pseudo pocket or gingival pocket, whereas the apical migration of the junctional epithelium with the destruction of supporting periodontal tissues is responsible for developing a true pocket or periodontal pocket [3].

Non-surgical periodontal therapy is considered as a “golden standard” treatment protocol for periodontal pockets because it involves scaling and root planing (SRP) to remove supra and subgingival biofilms, plaque, and calculus from diseased root surfaces [4,5]. It is proven that the non-surgical approach is an effective treatment strategy with periodontal pockets measuring less than the critical probing depth of 2.9 mm. However, in cases of deep periodontal pockets, surgical periodontal therapy along with periodontal regeneration stands to be a viable option, as complete debridement of biofilm remains crucial due to the presence of extensive periodontal tissue destruction.

Periodontal regeneration involves the use of various biomaterials like autogenous bone grafts [6], demineralized freeze-dried bone graft [7], xenografts [8,9], barrier membranes and different types of bone grafts [10], enamel matrix derivatives [11], platelet-rich fibrins [12,13], calcium sulphate [14], chitosan [15], and three-dimensional hydrogels (collagen, chitosan, hyaluronic acid-based) [16] combined with surgical procedures. Autologous platelet concentrates are effective, along with other regenerative materials, or alone in the treatment of intra-bony defects, furcation defects, and alveolar sockets [17,18]. Recent periodontal regenerative therapies using leukocyte- platelet-rich fibrin added to autogenous bone grafts [19] or inorganic bovine [9] showed favorable effects in hard and soft tissue regeneration.

Periodontal pockets present an ideal site for treatment using localized drug delivery systems. Within the field of periodontics, there are established approaches for delivering drugs directly to subgingival sites or periodontal pockets. These systems release antimicrobial agents either instantly or in a controlled and sustained manner, effectively countering microbial threats while minimizing potential adverse effects on non-oral parts of the body. Currently, a range of local drug delivery systems are available, including fibers, gels, strips, films, irrigating systems, and microparticles [20,21,22].

Propolis is a natural, non-toxic, resinous, yellow–brown to dark brown substance collected through honey bees from trees, buds, sap flow, shrubs, and other plant sources [23,24]. Propolis has various beneficial properties like antimicrobial, anticancer, antifungal, antiviral, and anti-inflammatory effects [25,26,27]. Propolis has been used extensively for the treatment of periodontitis in various forms such as paste, tablet, ointment, oil, mouthwash, etc. [28,29,30,31,32,33,34,35,36,37,38], or in patients undergoing oral surgical procedures where oral hygiene might have been impaired [39,40,41]. Propolis has also been used sub-gingivally for the management of periodontal pocket in patients with periodontitis [42,43] and has been found to be very effective.

This study aimed and was designed to explore the direct subgingival delivery of propolis nanoparticles into the periodontal pocket, serving as a local drug delivery (LDD) agent for the non-surgical management of periodontal pockets.

## 2. Materials and Methods

The present study was a prospective, double-blind, randomized clinical trial of parallel design (one site/patient) conducted among patients that visited the outpatient Department of Periodontics and Oral Implantology, Institute of Dental Sciences, SUM hospital, Siksha ‘O’Anusandhan (Deemed to be University), Bhubaneswar, Odisha. The ethical committee of IMS and SUM Hospital, SOA (Deemed to be University) approved the study with Ethical Number: Ref. no/IEC/IMS.SH/SOA/2022/418. This study strictly followed the CONSORT guidelines and was in accordance with the Declaration of Helsinki.

Patients were recruited based on the following inclusion and exclusion criteria.

Systemically healthy patients within the age group of 18–65 years, showing an acceptable oral hygiene with plaque scores of less than 1.5, who were diagnosed with generalized periodontitis of stages II and III (According to World Workshop of Periodontology, 2017 [44,45]) with at least one periodontal pocket with probing pocket depth of between 4 and 6 mm were included in the study. Smokers; pregnant and lactating mothers; patients who are allergic to propolis products; patients who received radiotherapy, chemotherapy or immunosuppressive treatments, systemic corticosteroids, and/or anticoagulants 30 days prior to intervention; patients with any systemic disease; and patients under the influence of non-steroidal anti-inflammatory drugs (NSAIDS) or any other anti-inflammatory medications, antibiotics, steroids that can alter the course of periodontitis progression and treatment were excluded.

### 2.1. Preparation Protocol of Propolis Nanoparticle as Liquid Solution and Application

Indian honey bee propolis was collected from Bhubaneswar, Odisha in November 2021. It then went through a cold-water washing process to remove the wax. The remaining part was dried, ground, and stored at 8 °C for further use. Propolis nanoparticle solution was prepared at room temperature by dissolving 0.22 g ground and dried propolis in 20 mL Milli-Q water, i.e., equivalent to 1.08 wt.%, using a 0.5-inch probe sonicator (Q500 Sonicator^®^, Qsonica, Newtown, CT, USA) for 4 h at room temperature, 300 K. The probe tip size used was 1 cm and the sonication depth was approximately 2 cm. The amplitude and intensity of sonication were set at 50% and 40 W, respectively. Then, the solution was filtered using Whatman 40 filter paper and extract was part stored at 8 °C for further applications. The propolis nanoparticle solution was sub-gingivally delivered inside the periodontal pocket and pocket was sealed using cyano-acrylate.

The size distribution of the propolis nanoparticles was determined using dynamic light scattering (DLS) analysis (Zeta-sizer Ultra, Malvern Panalytical, Malvern, UK). This technique measures the intensity-based size distribution of particles in a suspension. The results revealed a mean particle size with a range of 88.6 to 103 nanometers in diameter, with the largest particle size of 140 nm in diameter. The zeta potential of the propolis nanoparticles was assessed using zeta potential analysis (Zeta-sizer Ultra, Malvern Panalytical, Malvern, UK). This technique measures the electrostatic charge present on the surface of the particles. The results demonstrated a zeta potential of −20.51 mV, indicating the stability of the nanoparticles.

**Figure 1:**
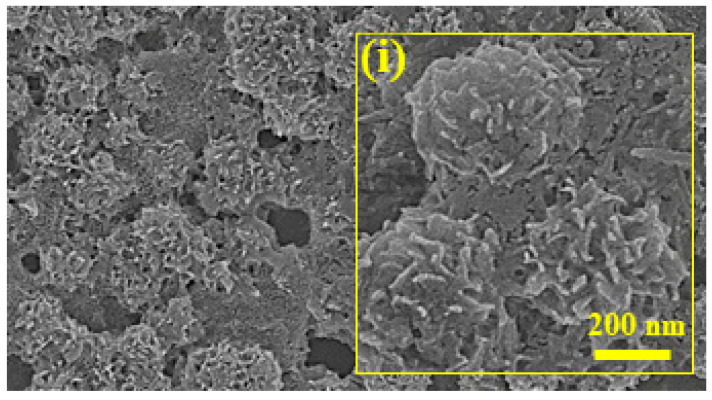
Characterization of propolis nanoparticles using FE-SEM, mag. 20,000×; (i) flower-shaped nano-particle, mag. 50,000×.

The morphology of the propolis nanoparticles was examined through field emission scanning electron microscopy (FE-SEM) (Merlin Compact, Carl Zeiss, Jena, Germany). Micrographs obtained from FE-SEM analysis showed flower-shaped nanoparticles with a relatively uniform size distribution with sizes ranging between 88.6 and 103 nm with a structural diameter of less than 100 nm (Figure 1).

### 2.2. Biocompatibility of Propolis Nanoparticle

**Figure A1:**
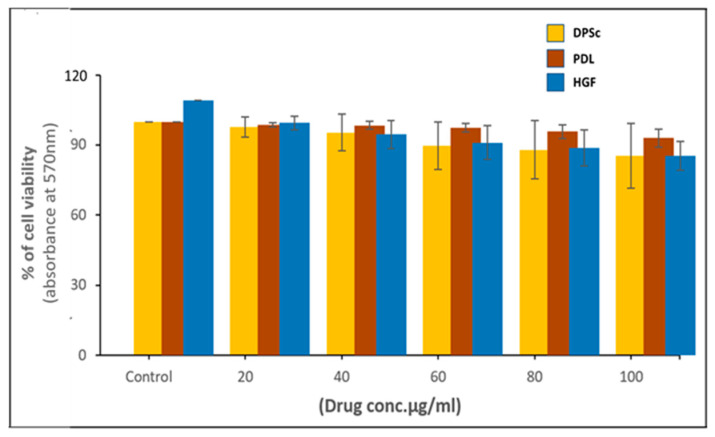
The cell viability of control and experimental groups was tested by MTT assay for 24 h, respectively, and absorbance was taken at 570 nm. Data are shown as means ± SD (*n* = 3), *p* < 0.001.

The biocompatibility of the obtained nanoparticle was carried out using MTT assay. The details can be found in Appendix A (Figure A1).

### 2.3. Study Groups

The selected sites of all patients were marked and assigned randomly either to the control group or test group.

Test Group (SRP + PRO) (*n* = 20): sites treated with SRP followed by sub-gingival delivery of propolis nanoparticle.

Control Group (SRP + Saline) (*n* = 20): sites treated with SRP followed by sub-gingival delivery of saline (placebo).

#### 2.3.1. Randomization and Blinding

The randomization was carried out using computer generated table of random numbers. The allocated groups were concealed using opaque sealed envelopes, which were opened prior to the sub-gingival delivery of LDD propolis nanoparticle or saline. The study was double-blind, where both the participants and investigator were unaware of the substance delivered.

#### 2.3.2. Patient Examination

On their first visit, all patients were clinically examined and the following indices were recorded: plaque index (PI) (site specific plaque index by Löe and Silness in 1967 [46]), gingival index (GI) (Löe and Silness, 1963 [47]), sulcus bleeding index (BI) (Muhlemann and Son, 1971 [48]), probing pocket depth (PPD), and relative attachment level (CAL). These indices or outcomes were measured at all six surfaces of the selected tooth (mesio-buccal, mid-buccal, disto-buccal, mesio-lingual, mid-lingual, and disto-lingual). These parameters were recorded at one month and three months after therapy.

#### 2.3.3. Technique for Sub-Gingival Delivery of Propolis

After baseline examination, test sites were treated with SRP followed by subgingival administration of propolis nanoparticle in the liquid solution form through blunt cannula, and control sites were treated with SRP followed by subgingival administration of saline as a placebo through a blunt cannula. The pockets were sealed using isoamyl-2-cyanoacrylate (Amcrylate). Clinical parameters were assessed at one month and three months after therapy.

### 2.4. Statistical Analysis

A sample size of 40 subjects and 20 subjects in each group were estimated to achieve 80% power in a two-tailed comparative test between the groups (*p* ≤ 0.05) by considering the mean difference of RAL 1.0 mm to be statistically significant between the test and control groups. The collected data were analyzed by using Statistical Package for Social Sciences (SPSS) software version 17.0. The data were tested for normality using Kolmogorov–Smirnov test and Shapiro–Wilk test. Mann–Whitney U test was employed to compare the data between two groups for inter-group comparison. Value of *p* ≤ 0.05 was considered to be statistically significant and *p* < 0.001 was considered highly statistically significant.

## 3. Results

**Figure 2:**
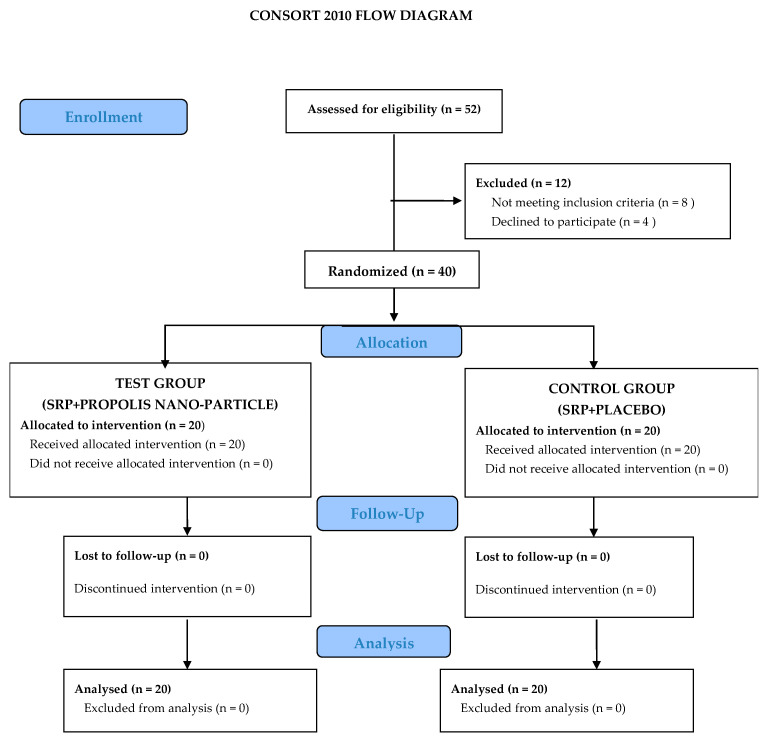
CONSORT flow chart showing allocation of sites.

A total of 52 subjects were screened for this study. Out of fifty-two subjects, eight did not meet the inclusion criteria and four refused to participate in this randomized controlled clinical trial. Therefore, 40 patients (10 females, 30 males) finally participated in this study. The patients were in the age group 18–65 years with probing pocket depths between 4 and 6 mm (Generalized periodontitis patients of stage II and III according to AAP classification, 2017 [45]). All the participants completed the study and there were no dropouts (Figure 2).

The data collected was subjected to statistical analysis using SPSS software, version 17.0. The *p*-value was set as 0.05 to represent statistical significance. The distribution of treated tooth of each subject and demographic details between two groups are provided in Table 1 and Table 2.

No significant differences between the groups were observed for plaque index at 1-month and 3-month time interval with *p* value equal to 0.429. The gingival index at 1 month and 3 months was found to be significantly better in SRP + PRO group than the SRP + Saline group with *p* value of <0.001 (Table 3).

The BOP, PPD, and RAL showed significant improvement with the SRP + PRO group at the end of 3 months follow-up with *p* values of 0.0001, 0.001, and 0.05, respectively (Table 4).

The change in all clinical parameters like GI, BOP, PPD, and RAL was found to be significantly better with the SRP + PRO group compared with the SRP + Saline group, except for PI with *p* value of 0.253 at the end of 1 month. However, change in PI at the end of 3 months was shown to be significantly better with the SRP + PRO group (Table 5).

## 4. Discussion

Propolis is a natural resinous substance collected by honeybees from tree buds, sap flows, and other botanical sources [49,50]. It has been used for its medicinal properties for thousands of years, and recent studies have shown that propolis contains various bioactive compounds, including flavonoids, phenolic acids, terpenes, and esters. Propolis has been found to have antibacterial, antifungal, antiviral, anti-inflammatory, and antioxidant properties, making it a promising agent for the treatment of periodontal pockets [51,52,53,54,55,56,57].

Propolis has also been used as a promising agent in the treatment of periodontal disease, like the use of PRF in regenerative dentistry, and shows promising results in soft tissue as well as hard tissue healing [13,17,18,58,59,60,61,62,63,64,65]. Probiotics also play an important role in the non-surgical management of periodontitis [66]. In this study, the use of propolis nanoparticles in the non-surgical treatment of periodontal pockets showed similar promising results.

However, several contributing factors may affect treatment outcome. Poor oral hygiene is detrimental and maintaining a good oral hygiene is essential for preventing various oral diseases, including gingivitis, periodontitis, and bad breath [67]. Another contributing factor is the use of certain medications. Some medications, such as antihistamines, antidepressants, and antihypertensive drugs, can cause dry mouth, which reduces saliva flow and increases the risk of oral health problems [68,69,70]. Similarly, individuals who use tobacco products or consume alcohol excessively may have a higher risk of developing oral diseases due to the detrimental effects of these substances on oral health [71,72]. Poor dietary habits can also affect oral hygiene. A diet high in sugar and processed foods can increase the risk of dental caries, while a diet lacking in essential nutrients, such as calcium and vitamin D, can lead to weakened teeth and gums [73,74,75,76,77,78,79,80,81]. Lastly, mental health and stress levels can also play a role in maintaining oral hygiene, which can cause damage to the teeth and gums over time [82,83,84,85,86,87,88].

Scaling and root planing is a non-surgical periodontal therapy that is commonly used to treat patients with periodontitis [89]. In this study, ultrasonic scaling and root planing was used to perform mechanical debridement in patients suffering from chronic periodontitis. Overall, both ultrasonic and manual methods of mechanical debridement are effective for subgingival instrumentation, and the choice between them may depend on factors such as the severity of the patient’s periodontitis and their level of discomfort [89].

In this study, the use of Indian propolis in the form of nanoparticles for the treatment of periodontal pockets showed promising results. Other studies have investigated the beneficial effects of propolis from different plant sources on periodontal treatment outcomes and the beneficial effects of propolis may be based on the source of origin related to the geographical area. Recently, propolis has been used in various formulations for the treatment of periodontitis because of some disadvantages of raw propolis, such as strong and unpleasant taste, strong aromatic smell, and high ethanol concentration [57,90]. So, the application of microparticles can improve and increase the therapeutic effect from biomedical materials or from drugs. However, the microparticles did not show activity against all the tested strains until the concentration of 0.30 mg/mL [91], but in other study, propolis showed *in vitro* antimicrobial activity against all the tested microorganism strains [92]. Recently, propolis nanoparticles (a size of less than 100 nanometers) have gained attention as a potential therapeutic agent for periodontitis due to their unique physicochemical properties, such as high surface area-to-volume ratio and increased bioavailability. This study suggests that propolis nanoparticles have potential as a natural and effective treatment option for periodontal pockets, but further research is needed to fully understand their mechanisms of action and optimize their formulation and delivery for clinical use.

Biocompatibility testing is essential in determining the safety and effectiveness of any dental material, including propolis, before its clinical use. It has been investigated in several studies on human gingival fibroblasts (HGF), dental pulp stem cells (DPSCs), and periodontal ligament (PDL) cells, which have suggested that propolis extracts may have potential for use in periodontal treatment [93,94,95]. In this study, the cell viability of propolis nanoparticles was tested using MTT assay for 24 h and >80% cell viability was present at 100% concentration in HGF, DPSCs, and PDL cells. Our study revealed that propolis nanoparticles exhibited a greater inhibition of DPSCs and HGF compared with PDL cells. It is important to note that our *in vitro* study evaluated the effects of propolis nanoparticles on isolated cell cultures, which may not fully replicate the complex microenvironment of the gum tissue *in vivo*.

In this study, the change in all the clinical parameters like GI, BOP, PPD, and RAL was found to be significantly better with the SRP + PRO group compared with the SRP + Saline group at end of the 1-month and 3-month time-interval. Similar results were obtained by previous studies which used other active agents like probiotics [96], chlorhexidine [97], PRGF [13], and tea-tree oil [98] as subgingival delivery or intra-pocket delivery agents for the management of periodontal pockets at a 3-month time interval.

Propolis has been used extensively for the treatment of periodontitis in various forms such as toothpaste [31,34,35] and mouthwash [29,32,35] due to its various beneficial properties like antimicrobial, anticancer, antifungal, antiviral, and anti-inflammatory effects [25,26,27]. Several studies have investigated the effectiveness of the subgingival irrigation of propolis for the treatment of periodontitis and found that propolis irrigation improves clinical parameters as well as significantly reduces the number of bacteria in periodontal pockets, particularly anaerobic bacteria that are associated with periodontitis. In the present study, a significant increase in PPD and RAL was observed at the 3-month recall check-up in both the groups but more gain was seen in test group sites along with improvements in other clinical and microbiological parameters, which shows the efficacy of propolis nanoparticles as local drug delivery in the form of a liquid solution over SRP alone.

In periodontics, various tissue adhesives have been investigated for the closure of periodontal pockets [99]. The use of fibrin and cyanoacrylate glue showed fast control over bleeding, easy to apply and beneficial in terms of patient’s acceptance, especially in periodontal surgeries [100,101,102]. In this study, isoamyl 2-cyanocrylatec (Bio-adhesive) was used to seal periodontal pockets after the placement of a propolis nanoparticle as a local drug delivery system for the sustained release of the entrapped drug into the periodontal pocket.

The current study is a randomized controlled trial, with a parallel design, comparing the use of the intra-pocket delivery of propolis nanoparticles. Split-mouth design is considered to be ethically sound as each patient receives both the experimental and control interventions [103]. A split-mouth study design to evaluate the efficacy of propolis nanoparticles may be a future concern.

The decision to exclude stage IV periodontitis patients from our study was based on several factors. Firstly, stage IV periodontitis is characterized by severe periodontal tissue destruction, including extensive bone loss and tooth mobility. These patients often require more complex and aggressive treatment approaches, such as surgical interventions and advanced periodontal therapies. Our study, on the other hand, focused on assessing the effectiveness of a specific non-surgical intervention in early to moderate stages of periodontitis.

Including stage IV patients would have introduced significant heterogeneity to the study population, with varying treatment needs and potential confounding factors that could impact the outcomes. It is important to note that the exclusion of these patients was not meant to undermine the significance of their condition, but rather to maintain a homogeneous study population with similar baseline characteristics and treatment requirements.

Propolis is generally considered safe and well-tolerated. However, propolis as a natural product may contain environmental pollutants including heavy metals [104]. There was no adverse effect seen in any of the patients treated with propolis in the present study. The various benefits of propolis such as affordability, easy availability, and antibacterial and anti-inflammatory properties, make propolis a potential therapeutic agent in periodontal therapy. The strength of this study lies in its rigorous study methodology of treating one site per patient to avoid the confounding bias of pocket recolonization and being the first study of its kind to evaluate the effects of propolis nanoparticles in the non-surgical management of periodontal pockets. The limitations of the present study include a small sample size, a shorter follow-up time limited to a maximum of 3 months, and a lack of biomarker test. Considering the confounding variables, such as the severity of periodontal disease at baseline, the patients’ oral hygiene practices, and their compliance with treatment protocols, could have impacted the response to the propolis nanoparticle intervention. Future research is warranted to consider these factors.

## 5. Conclusions

In conclusion, our study demonstrates the potential of propolis nanoparticles as a natural and effective treatment option for periodontal pockets. The sub-gingival administration of propolis nanoparticles with scaling and root planing resulted in significant improvements in various periodontal parameters, including reductions in GI, BOP, PPD, and RAL compared with the control sites treated with saline and SRP.

The subgingival delivery of propolis nanoparticles as an addition to scaling and root planing holds promise for the treatment of chronic periodontitis. Future investigations should explore the effects of higher concentrations of propolis extract and increased frequency of application, as they may potentially yield even more favorable outcomes. Moreover, additional research with larger sample sizes and longer observation periods is necessary to gain a comprehensive understanding of the mechanisms underlying propolis nanoparticles’ action, optimize their formulation and delivery, and establish their safety and efficacy in human subjects.

Overall, propolis nanoparticles exhibit encouraging potential as a natural therapeutic approach for managing periodontal pockets. Continued scientific inquiry and clinical trials will contribute to further refining this treatment modality and expanding its application in the field of periodontics.

## Figures and Tables

**Table 1:** Distribution of sites in test and control group.

Teeth	Group		Total
	A: SRP + PRO ^1^	B: SRP + SALINE ^2^	
Incisor	4	7	11
Canine	4	3	7
Premolar	3	4	7
Molar	9	6	15
Total	20	20	40

^1^ SRP + PRO = scaling and root planing followed by Propolis delivery; ^2^ SRP + SALINE = scaling and root planing followed by saline delivery.

**Table 2:** Distribution of age in test and control group.

Treatment Group	No. of Patient	Mean	Standard Deviation	Mean Standard Error
SRP + PRO	20	49.90	11.625	2.599
SRP + Saline	20	50.10	12.208	2.730

**Table 3:** Intergroup comparison of PI and GI at different time intervals in both groups.

Parameter	Groups	N	Median	Max.	Min.	Average Rank	Sum of Ranks	*p*-Value
PI_Baseline	SRP + PRO	20	1.6000	2.0000	1.2500	21.50	430.00	0.602
	SRP + Saline	20	1.5500	1.6250	1.2500	19.50	390.00	
	Total	40						
PI_1months	SRP + PRO	20	0.5625	1.0000	0.2500	19.03	380.50	0.429
	SRP + Saline	20	0.6375	0.7500	0.2500	21.98	439.50	
	Total	40						
PI_3months	SRP + PRO	20	0.6750	1.2500	0.2500	16.88	337.50	0.429
	SRP + Saline	20	0.8875	1.0000	0.5000	24.13	482.50	
	Total	40						
GI_baseline	SRP + PRO	20	1.7500	2.0000	1.5000	20.50	410.00	1.000
	SRP + Saline	20	1.7500	1.7500	1.5000	20.50	410.00	
	Total	40						
GI_1months	SRP + PRO	20	0.7250	1.2500	0.5000	13.40	268.00	* 0.000
	SRP + Saline	20	1.0375	1.0000	0.5000	27.60	552.00	
	Total	40						
GI_3months	SRP + PRO	20	0.3250	1.0000	0.2500	14.40	288.00	* 0.001
	SRP + Saline	20	0.5625	0.5000	0.2500	26.60	532.00	
	Total	40						

* Mann-Whitney U Test *p* < 0.05 statistically significant.

**Table 4:** Intergroup comparison of BOP, PPD, and RAL at different time intervals in both groups.

Parameter	Groups	N	Median	Max.	Min.	Average Rank	Sum of Ranks	*p*-Value
BOP_Baseline	SRP + PRO	20	2.4625	3.0000	2.0000	21.18	423.50	0.718
	SRP + Saline	20	2.4250	2.5000	2.0000	19.83	396.50	
	Total	40						
BOP_1months	SRP + PRO	20	0.4375	0.5000	0.0000	20.95	305.70	0.718
	SRP + Saline	20	0.5875	0.5000	0.0000	18.93	408.50	
	Total	40						
BOP_3months	SRP + PRO	20	0.1625	0.2500	0.0000	12.95	259.00	* 0.000
	SRP + Saline	20	0.4500	0.2500	0.0000	28.05	561.00	
	Total	40						
PPD_baseline	SRP + PRO	20	4.9000	6.6500	4.0000	20.50	410.00	1.000
	SRP + Saline	20	4.9000	6.0000	4.0000	20.50	410.00	
	Total	40						
PPD_1months	SRP + PRO	20	2.9000	4.6500	2.0000	17.65	353.00	0.127
	SRP + Saline	20	3.3000	5.0000	3.0000	23.35	467.00	
	Total	40						
PPD_3months	SRP + PRO	20	2.4500	3.2000	2.0000	14.33	286.50	* 0.001
	SRP + Saline	20	3.1500	3.0000	2.0000	26.68	533.50	
	Total	40						
RAL_Baseline	SRP + PRO	20	9.6500	11.3000	8.9500	21.08	421.50	0.758
	SRP + Saline	20	9.5500	11.5500	8.0000	19.93	398.50	
	Total	40						
RAL_1months	SRP + PRO	20	7.6500	6.5500	9.9500	19.10	382.00	0.451
	SRP + Saline	20	7.9500	10.0000	6.0000	21.90	438.00	
	Total	40						
RAL_3months	SRP + PRO	20	7.2000	9.9500	6.7500	16.90	338.00	* 0.05
	SRP + Saline	20	7.8000	9.0000	6.0000	24.10	482.00	
	Total	40						

* Mann–Whitney U Test *p* < 0.05 statistically significant.

**Table 5:** Change in parameters at different time intervals in both groups.

Parameter	Groups	N	Median	Max.	Min.	Average Rank	Sum of Ranks	*p*-Value
ChangePI_1month	SRP + PRO	20	−1.0375	−0.25	−1.50	18.35	367.00	0.253
	SRP + Saline	20	−0.9125	−0.25	−1.50	22.65	453.00	
	Total	40						
ChangePI_3month	SRP + PRO	20	−0.9250	−0.25	−1.50	16.30	326.00	* 0.023
	SRP + Saline	20	−0.6625	−0.00	−1.20	24.70	494.00	
	Total	40						
ChangeGI_1month	SRP + PRO	20	−1.0250	−0.50	−1.25	14.68	293.50	* 0.001
	SRP + Saline	20	−0.7125	−0.25	−1.25	26.33	526.50	
	Total	40						
ChangeGI_3month	SRP + PRO	20	−1.4250	−1.00	−1.75	15.40	308.00	* 0.005
	SRP + Saline	20	−1.25	−1.00	−1.5	25.60	512.00	
	Total	40						
ChangeBOP_1month	SRP + PRO	20	−2.0250	−1.75	−2.25	14.35	287.00	* 0.001
	SRP + Saline	20	−1.8375	−1.75	−2.25	26.65	533.00	
	Total	40						
ChangeBOP_3month	SRP + PRO	20	−2.3000	−2.00	−2.75	13.75	275.00	* 0.000
	SRP + Saline	20	−1.9750	−1.75	−2.25	27.25	545.00	
	Total	40						
ChangePPD_1month	SRP + PRO	20	−2.0000	−1.00	−3.00	16.90	338.00	* 0.052
	SRP + Saline	20	−1.6000	−1.00	−3.00	24.10	482.00	
	Total	40						
ChangePPD_3month	SRP + PRO	20	−2.4500	−1.00	−4.00	15.48	309.50	* 0.006
	SRP + Saline	20	−1.7500	−1.00	−3.00	25.53	510.50	
	Total	40						
ChangeRAL_1month	SRP + PRO	20	−2.7500	−1.00	−4.00	16.90	338.00	* 0.052
	SRP + Saline	20	−1.7500	−1.00	−3.00	24.10	482.00	
	Total	40						
ChangeRAL_3month	SRP + PRO	20				15.48	309.50	* 0.006
	SRP + Saline	20				25.53	510.50	
	Total	40						

* Mann-Whitney U Test *p* < 0.05 statistically significant.

## Data Availability

The data presented in this study are available on request from the corresponding author.

## Appendix A

The biocompatibility of the obtained nanoparticle was carried out using an MTT assay.

A1. Isolation of primary cells, Cell Culture, and Caffeic acid phenethyl ester (CAPE) Treatment

Primary cells like human gingival fibroblasts (HGF), dental pulp stem cells (DPSCc), and periodontal ligaments (PDL) cells were isolated through mechanical digestion and cultured in Dulbecco’s Modified Eagle’s Medium supplemented with a nutrient mixture, which comprised F-12 Ham’s medium (Life Technologies, Grand Island, NY, USA), 10% fetal bovine serum (Hyclone Laboratories, Logan, UT, USA), 2 mM glutamine,100 U/mL penicillin, and 100 μg/mL streptomycin. All cell cultures were maintained at 37 °C in a humidified atmosphere of 5% CO_2_. For CAPE treatment, appropriate amounts of stock solution of CAPE were added into a culture medium to achieve the indicated concentrations and then incubated with cells for indicated time periods, whereas dimethyl sulfoxide solution without CAPE was used as a blank reagent.

A2. Determination of Cell Viability (MTT Assay)

For the cell viability experiment, a microculture tetrazolium (3-(4,5-dimethylthiazol-2-yl)-2,5-diphenyltetrazolium bromide) colorimetric assay was performed to determine the cytotoxicity of CAPE. Primary cells were seeded in 24-well plates at a density of 5 × 10^4^ cells/well and treated with CAPE at a concentration between 0 and 40 μM at 37 °C for 24 and 48 h. After the exposure period, the media were removed, and cells were washed with phosphate-buffered saline (PBS) and then incubated with 20 μL MTT (5 mg/mL) (Sigma chemical Co., St. Louis, MO, USA) for 4 h. After MTT Assay, the experiment was under observation. Then, quantitative and qualitative analyses were performed using spectrophotometric and phase contrast microscopy methods, respectively. The viable cell number per dish was directly proportional to the production of formazan, which can be measured spectrophotometrically at 563 nm following solubilization with isopropanol.

The biocompatibility of the propolis nanoparticle was found to be high with more than 80% cell viability with HGF, DPSCc, and PDL cells at all given drug concentrations of the prepared solution compared with saline as a control (Figure A1).
